# MACSFeD—a database of mosquito acoustic communication and swarming features

**DOI:** 10.1093/database/baae086

**Published:** 2024-08-28

**Authors:** YuMin M Loh, Matthew P Su, Kayla G Haruni, Azusa Kamikouchi

**Affiliations:** Graduate School of Science, Nagoya University, Nagoya 464-8602, Japan; Institute of Transformative Bio-Molecules (WPI-ITbM), Nagoya University, Nagoya 464-8601, Japan; Graduate School of Science, Nagoya University, Nagoya 464-8602, Japan; Institute of Transformative Bio-Molecules (WPI-ITbM), Nagoya University, Nagoya 464-8601, Japan; Institute for Advanced Research, Nagoya University, Nagoya 464-8601, Japan; Graduate School of Science, Nagoya University, Nagoya 464-8602, Japan; Graduate School of Science, Nagoya University, Nagoya 464-8602, Japan; Institute of Transformative Bio-Molecules (WPI-ITbM), Nagoya University, Nagoya 464-8601, Japan

## Abstract

Acoustic communication plays an important role during the courtship of many mosquito species. Male mosquitoes show strong attraction to female wing beat frequencies, mediated via spectral matching between female wing beat frequency and male ear mechanical tuning frequency. Such acoustic communication typically occurs within swarms, male-dominated aggregations with species-specific properties. Despite hundreds of relevant publications being available, the lack of a central platform hosting all associated data hinders research efforts and limits cross-species comparisons. Here, we introduce MACSFeD (Mosquito Acoustic Communication and Swarming Features Database), an interactive platform for the exploration of our comprehensive database containing 251 unique reports focusing on different aspects of mosquito acoustic communication, including hearing function, wing beat frequency and phonotaxis, as well as male swarming parameters. MACSFeD serves as an easily accessible, efficient, and robust data visualization tool for mosquito acoustic communication research. We envision that further in-depth studies could arise following the use of this new platform.

**Database URL**: https://minmatt.shinyapps.io/MACSFeD/

## Introduction

Almost half of the world’s population is at risk of infection from mosquito-borne diseases, such as dengue, Zika, and malaria, placing a significant socioeconomic burden on many countries [1, 2]. These diseases are predominantly spread by vectors from three mosquito genera; namely, *Aedes, Anopheles*, and *Culex* [3]. Due to the lack of effective therapeutics, current disease control programs heavily rely on insecticide-based methods that directly target the vectors themselves, such as long-lasting insecticide-treated nets and indoor residual spraying [4]. However, the widespread use of insecticides has resulted in the rapid development of insecticide resistance among mosquito populations, making new control tools urgently needed [5].

One promising alternative control strategy is to target and manipulate the mating behaviors of disease-transmitting mosquitoes [6]. Mating typically takes place in transient swarms consisting of large numbers of flying males [7]. Within these swarms, acoustic communication between male and female mosquitoes is a crucial precopulatory component that determines the outcome of courtship events [8]. An example of such acoustic communication is male phonotaxis—the attraction of male mosquitoes to conspecific female wing beat frequencies (WBFs) [9]. Sexual dimorphisms in mosquito WBFs enable males to accurately discriminate female sounds from their own [10].

Hearing behaviors rely on hearing function. Mosquito ears consist of two major components, a flagellum that vibrates in response to particle bombardment and a Johnston’s organ (JO) composed of auditory neurons responsible for converting such mechanical vibrations into electrical signals that are ultimately relayed to the brain [11, 12]. The mosquito JO is the largest in the insect kingdom and contains a complex auditory efferent network, facilitating highly sensitive hearing function [10, 13]. In some species, spectral matching between the mechanical vibrations of male ears (male ear mechanical tuning frequency) and female WBFs reflects a system in-tuned across several auditory components, with the male JO neuron response properties (male ear electrical tuning) mediating such matching [14].

Previous reports have identified interspecific differences in terms of maximally sensitive tuning frequencies of male ear at both mechanical and electrical levels, with researchers therefore suggesting that male mosquitoes may utilize a distortion product-based acoustic communication system in which the male auditory neurons are most sensitive to acoustic distortion products arising from the nonlinear interference of male and female WBFs [15]. Mosquito acoustic communication therefore relies on three interlinked, core components—male and female WBFs, male ear tuning frequencies, and male phonotaxis [8].

Extensive research has been conducted into each of these topics individually, in part to identify and guide the development of novel methods for mosquito control. This includes sound traps that play back female WBFs to lure and capture male mosquitoes or targeted spraying of swarms using insecticides [7, 16]. A comprehensive understanding of male swarming and courtship behaviors, and the implicated acoustic communication systems, could help inform the development of temporal-, spatial-, niche-, and species-specific mosquito control tools [8]. Such understanding is also essential for guiding the design and deployment of reproductive control tools that rely on mating for successful dissemination in the wild [17–19].

Furthermore, while for most disease-transmitting mosquito species only males demonstrate phonotaxis, in non-human biting species female phonotaxis has also been reported [20, 21]. Female *Uranotaenia* mosquitoes, which obtain blood meals from frogs, show positive phonotaxis behaviors towards frog calls [20, 21]. From a basic science perspective, exploring species-specific differences in the hearing systems of mosquitoes could shed light on how these systems have evolved to support sex- and species-specific behaviors [22, 23].

Despite the existence of a sizeable body of research, efforts to link these distinct components into a unified whole remain underwhelming, in part due to limited access to the vast body of available literature. Here, we introduce MACSFeD (Mosquito Acoustic Communication and Swarming Features Database), an interactive platform that provides access to a total of 251 collection records related to mosquitoes (Culicidae), which spans four major topics: swarming, WBFs, hearing function, and behavior. It is designed to provide a platform for easy amalgamation and visualization of the vast body of published literature regarding the hearing systems and swarming behaviors of mosquitoes, facilitating sex and species comparisons on topics of interest.

MACSFeD can be accessed via https://minmatt.shinyapps.io/MACSFeD/, with associated information for each dataset freely available via Mendeley Data (https://doi.org/10.17632/gcjtzfv4s3.1) and GitHub (https://github.com/MatthewPaulSu/MACSFeD_data). MACSFeD will be regularly updated with the release of new papers, but we welcome users/researchers to provide new information via the Google Forms link included in the platform. The website is free and open access to all researchers and public users who are interested in understanding the implications of mosquito swarming and hearing systems on their mating and reproductive biology.

## Materials and methods

### Literature selection

Different electronic databases were utilized to identify articles associated with the following topics: male swarming, male/female WBFs, male/female hearing function (mechanical/electrical tuning of the flagellar ear), and male/female (positive) phonotaxis behavior of mosquitoes (Culicidae) of any species. The first round of searching was performed during October 2021, and updated searches were conducted during December 2023 and June 2024. We searched for relevant peer-reviewed publications on PubMed while Scopus, Web of Science, Google Scholar, and OpenGrey were used to further identify gray literature. Non-peer reviewed articles identified from preprint servers such as bioRxiv were also included in the database. Article duplicates identified from different electronic databases were identified and removed using Zotero.

The following search terms were used for each search category in combination with the appropriate Boolean Operators and truncated (*) form of each keyword:

(1) Male swarming:

“swarm”, “lek”, “aggregation”, “mosquito”.

(2) Male/female WBFs:

“flight tone”, “wing beat”, “sound”, “frequency”, “mosquito”.

(3) Male/female hearing function:

“pedicel”, “Johnston’s organ”, “chordotonal organ”, “scolopidium”, “ear”, “antennae”, “flagellum”, “hearing”, “audition”, “mechanical”, “electrical”, “tuning”, “compound action potential”, “bioacoustics”, “mosquito”.

(4) Male/female phonotaxis behavior:

“phonotaxis”, “attraction”, “sound”, “acoustic”, “trap”, “lure”, “mosquito”.

By utilizing the above search strategy, we identified and included research articles in our analyses without setting any language or date restrictions. For each round of screening, two reviewers independently conducted two consecutive rounds of screening; a first round of title and abstract screening followed by full-text screening to check the suitability of these articles. Any discrepancies in screening outcome were resolved via discussions between the two reviewers. The reference sections of these eligible articles were further screened to identify potential articles for inclusion. In total, 251 unique article entries were included in this database, with 124, 87, 32, and 41 unique papers identified for swarming, WBFs, hearing function, and phonotaxis, respectively.

### Data extraction

Data extraction for each of the four topics was conducted separately by two reviewers by reading and extracting target information from the full text of eligible articles. Note that for papers that performed measurements/conducted observations on non-wild-type mosquitoes or mosquitoes not tested under natural conditions (such as transgenic, *Wolbachia*/virus-infected or compound/radiation/fluorescent powder-treated mosquitoes), only wild-type or control-treated information was extracted if available. Review articles and descriptive studies that did not provide original numerical values were excluded from this analysis.

For swarming papers, information including the genus, species, experimental location, time of swarming, swarm initiation time, swarming duration, swarm size (number of males participating in a swarm), and swarming height above ground were extracted if reported.

For WBF, information including the genus, species, sex, experimental location, flight condition, temperature, and reported WBF values were extracted if possible.

For hearing function, information including the genus, species, sex, experimental location, stimulus type provided, temperature, and the mechanical state of the mosquito when tested were extracted if available.

Finally, for reports on male/female positive phonotaxis behaviors, we extracted the genus, species, sex, experimental location, stimulus type, and phonotaxis scoring method, if available. We also extracted temperature information for phonotaxis reports reporting male phonotactic attraction to different frequencies of sound.

All extracted data points were compiled in Excel and served as the database for building the MACSFeD data visualization tool. If an identified paper provided several measurements collected under different conditions, the measurement associated with each condition was extracted as an independent entry.

Due to the renaming/reclassification of certain species, *Culex fatigans* was referred to here as *Culex quinquefasciatus* [24] and the molecular M and S forms of *Anopheles gambiae (s.l.)* were grouped under *Anopheles coluzzii* and *Anopheles gambiae (s.s.)*, respectively [25].

To facilitate comparison of phonotactic profiles reported in different papers, the phonotactic responses of the tested group to each pure tone frequency (in absolute counts or response proportion/percentage) were normalized by dividing the response count for one pure tone to the largest response count within the same report. Thus, response counts were all converted and scaled into values in the range of 0 (no response to the indicated pure tone) to 1 (maximum response to the indicated pure tone among all pure tones tested in the indicated study). Due to the nature of such normalization, only papers that tested more than one pure tone frequency in their phonotaxis assays were included in the normalized frequency response plots.

For papers that reported measurements such as temperature and WBF in the form of a range of values instead of single values (i.e. mean or median values), the average of the endpoints of the range was computed and this single averaged value was included in the database. For data visualization purposes, mean and median values extracted were plotted together. For each of the plots presented in MACSFeD (apart from plots related to the number of reports or frequency vs temperature plots), the crossbar represents the median value of the indicated data group.

### User interface creation

MACSFeD was created using R Shiny (version 1.7.4) [26] and converted into HTML format using the shinylive (ver 0.1.1) [27] and httpuv (version 1.6.14) packages. The R packages ggplot2 (version 3.4.2) [28] and plotly (version 4.10.2) [26] were used to create all plots. The MACSFeD user interface was tested by researchers for functionality prior to being made publicly available.

## Results

### Database overview

**Figure 1.**
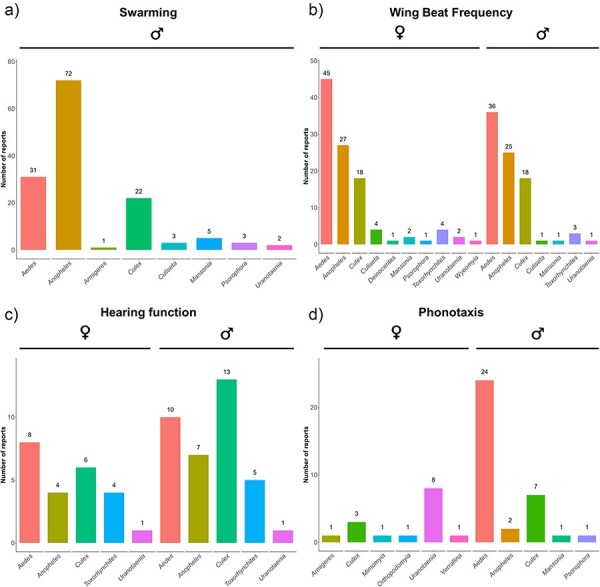
The number of unique reports per genus/sex for each of the four research a reas included in the database: (a) swarming, (b) WBF, (c) hearing function, and (d) phonotaxis.

The MACSFeD database includes literature reports covering four major data types describing the acoustic-related mating behaviors of mosquitoes; namely swarming, WBFs, hearing function, and phonotaxis behavior. The summary overview provided in Fig. 1 shows the number of unique publications for each data type, grouped by sex and mosquito genus. The database includes a total of 124, 87, 32, and 41 entries for swarming, WBF, hearing function, and phonotaxis behavior, respectively, with each data type containing information on multiple genera of mosquitoes. Individual papers may include data on either multiple data types, or multiple data points for an individual data type. As such, while the number of individual papers included is 251, 954 data points are included in the overall dataset.

**Figure 2.**
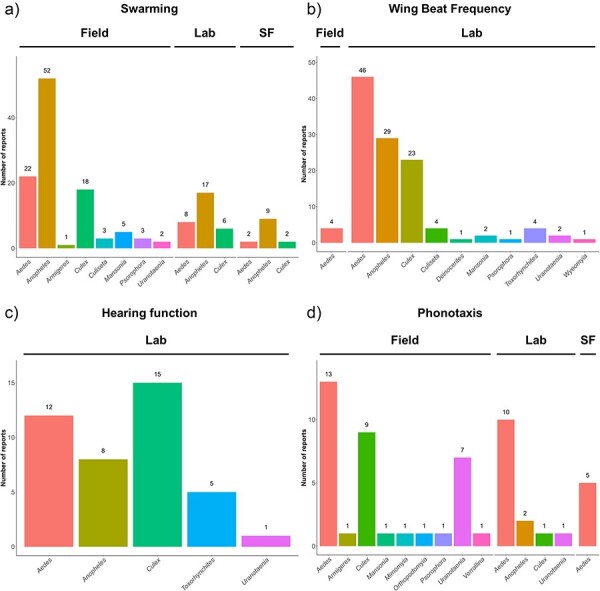
The number of unique reports per genus grouped based on the reported experimental location (field, lab, semi-field) for each of the four research areas: (a) swarming, (b) WBF, (c) hearing function, and (d) phonotaxis. SF = semi-field.

**Figure 3.**
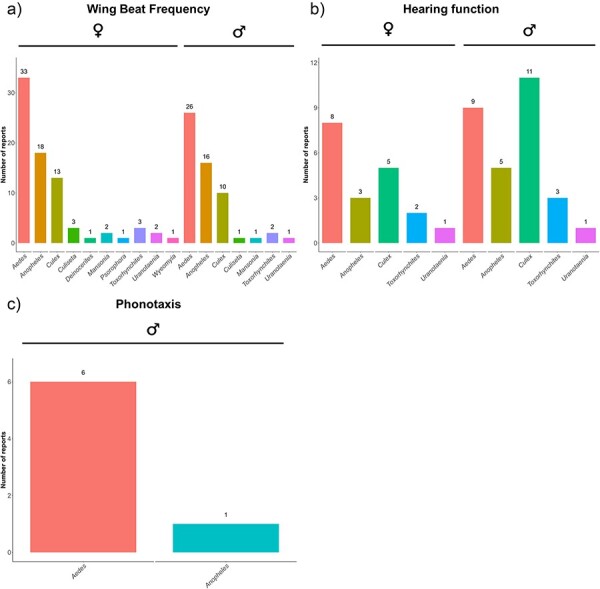
The number of unique reports per genus that included information on the experimental temperature for (a) WBF, (b) hearing function, and (c) male phonotactic responses to different frequencies of sound.

Apart from species-specific comparisons, our database also allows users to draw comparisons between sexes where data are available (Fig. 1). Since differences in experimental location may account for variations between data entries, potentially due to changes in the environmental conditions, we extracted the experimental location reported for each measurement where available (Fig. 2). We additionally extracted temperature information whenever possible for datasets associated with WBF, hearing function, and male phonotactic responses to different frequencies of sound (Fig. 3).

### MACSFeD user interface

**Figure 4.**
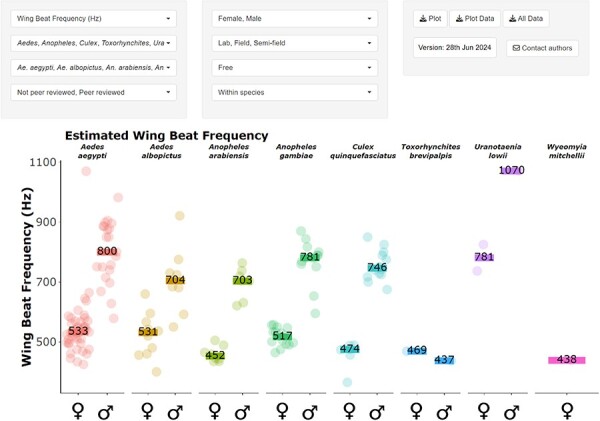
MACSFeD web interface overview showing interactive GUI.

MACSFeD is an interactive data visualization platform designed to provide users with direct access to our extensive literature database (Fig. 4). It is divided into four broad categories based on the data type: swarming, WBF, hearing function, and phonotaxis. Users can plot the number of reports associated with each data type. Data type-specific queries are also available under the “Data type” drop-down menu. Users can select to visualize only the genus and species of interest. The platform further provides a series of drop-down menus that allow users to customize the plot output based on the data properties/conditions such as mosquito sex and experimental location, with each of these selections applicable to all or a subset of data types. Cross-species or sex comparisons can be achieved by selecting the appropriate faceting option.

As temperature has been reported to be a key factor influencing the mosquito auditory system [29–31], we included an option which allows users to visualize the effect of temperature on WBF as well as ear mechanical and electrical tuning. For WBF or hearing function data plotted by temperature, the slope gradient is printed at the top of each panel. This gradient is calculated using a linear model (Frequency = gradient*Temperature + intercept) fit over the temperature range of all data points included by the user, with this model selected based on a previous report [29].

We incorporated interactive elements into our platform to allow users to obtain key information, such as the reported value and report title associated with each entry by hovering the cursor over each data point. A table will appear upon clicking a data point/bar, which includes relevant information such as hyperlinks directing users to the associated publications. The information contained in this table can be copied or saved as an Excel workbook.

As some reports in the database are from non-peer reviewed articles (such as theses available online), we further included an option allowing users to subset the data by peer review status.

Users can download plots using either plotly’s inbuilt screenshot feature or the “Download plot” option, with associated datasets available via the “Download plot data” button. Users can download the entire database by simply selecting the “Download all data” option. The complete database is available for download via Mendeley Data (https://doi.org/10.17632/gcjtzfv4s3.1) and GitHub (https://github.com/MatthewPaulSu/MACSFeD_data), along with all relevant code.

Lastly, to ensure the database keeps up to date with the latest findings, we encourage users to actively contribute toward the platform by clicking the “Contact authors” button to submit relevant information via the Google Forms link. This link also leads to a Frequently Asked Questions page.

### Case study

**Figure 5.**
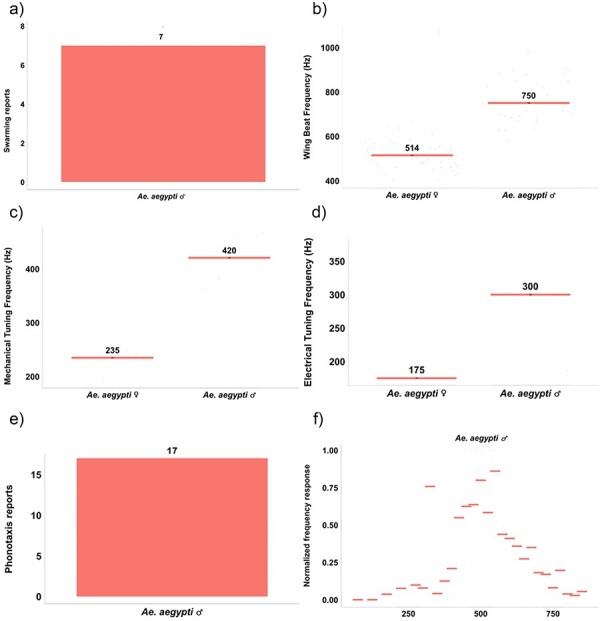
Case study of using MACSFeD to investigate previous literature studying the acoustic communication system of *Ae. aegypti*, including (a) number of unique reports documenting male *Ae. aegypti* swarming, (b) median of reported WBFs of female and male *Ae. aegypti*, (c) median of reported stimulated ear mechanical tuning frequencies of female and male *Ae. aegypti*, (d) median of reported stimulated ear electrical tuning frequencies of female and male *Ae. aegypti*, (e) number of unique reports describing the phonotaxis behavior of male *Ae. aegypti,* and (f) normalized phonotaxis frequency response profile of male *Ae. aegypti*.

MACSFeD allows users to directly contrast different elements of mosquito acoustic communication. To demonstrate potential comparisons users can draw, we extracted data related to *Aedes aegypti* (*Ae. aegypti*), a vector of dengue, Zika, and yellow fever viruses known to utilize hearing during mating (Fig. 5).

First, we searched the database for all prior reports on *Ae. aegypti* and identified seven papers detailing the male swarming behavior of this species (Fig. 5a). Next, we tested for sexual dimorphisms in WBFs and found estimated median values of ∼750 Hz for males and ∼515 Hz for females (Fig. 5b). Building on this, we checked for sexual dimorphisms in hearing function. For stimulated mosquito ear recordings, we found that the stimulated mechanical and electrical tuning frequencies of male *Ae. aegypti* have estimated medians of 420 Hz and 300 Hz, respectively, while for females these values were 235  and 175 Hz (Fig. 5c and d). In both sexes therefore electrical tuning values appear lower than mechanical tuning estimates.

Finally, we found 17 papers describing male *Ae. aegypti* phonotaxis behavior (Fig. 5e). Male *Ae. aegypti* have been reported to show attraction to pure tones between approximately 400  and 700 Hz, with peak attraction to tones of around 500 Hz, close to the WBFs of conspecific females (Fig. 5f). These analyses can be further refined by users interested in *Ae. aegypti* WBF data collected in specific conditions (such as free vs tethered flight).

## Discussion

Mosquito-borne diseases represent a global public health concern, making the development of novel control tools a priority. Hearing thus represents an ideal target for such novel tools, as hearing plays an important role in the mating behaviors of many medically relevant mosquito species. However, despite over a century of published research, mosquito auditory research is still hampered by the lack of a comprehensive database containing all available literature.

By amalgamating data across topics on MACSFeD, our database helps users to draw correlations between topics and identify knowledge gaps, thus facilitating the development and validation of testable hypotheses related to mosquito acoustic communication. By establishing this platform for mosquito auditory research, we hope to encourage mosquito acoustic communication researchers to include detailed information regarding experimental conditions when reporting new analyses; this includes experimental circadian time, temperature, humidity, age, experimental location, rearing conditions, tethering conditions, and equipment used.

Given the strong influence of abiotic factors on mosquito audition, such as temperature [29–31], future enrichment of the database with experiments focused on these parameters could help elucidate the contribution of different factors on mosquito hearing systems. For example, although estimating the influence of temperature on male electrical tuning is plausible using MACSFeD, equivalent data for females is too sparse to draw firm conclusions. Furthermore, while changes in male mosquito WBFs have been identified across the day [31], equivalent testing of potential circadian changes in hearing function has not been conducted. Most reports included in our database are centered on testing *Aedes, Anopheles*, or *Culex* mosquitoes; experiments centered on non-human biting mosquitoes are therefore of interest to facilitate more complex comparisons across different groups.

As CRISPR-Cas9-based mutagenesis techniques applicable to mosquitoes continue to be refined, the future will likely see a surge in genetically modified mosquitoes being generated and tested for mating fitness [32, 33]. We anticipate expanding the current database to include the auditory and swarming features of genetically engineered mosquitoes to provide a platform for researchers to explore the molecular mechanisms underlying mosquito acoustic communication. We also intend to link users from our platform to databases (such as Vectorbase [34]) that could provide detailed information about the genes of interest.

We hope to further expand MACSFeD to incorporate a broader range of data parameters which would allow for different types of data visualization depending on community input. In addition to reporting specific experimental conditions, we encourage researchers to share data analysis scripts and raw data after publication so that new data points can be incorporated rapidly into MACSFeD. We hope that MACSFeD can serve as a useful platform for understanding the different aspects of mosquito acoustic communication system, thereby contributing towards the identification of suitable hearing/swarming targets for vector control.

## Data Availability

MACSFeD can be accessed at: https://minmatt.shinyapps.io/MACSFeD/ All code for MACSFeD, as well as associated databases, is available at: https://doi.org/10.17632/gcjtzfv4s3.1 A back-up can be found at: https://github.com/MatthewPaulSu/MACSFeD_data To highlight database errors, or to request the inclusion of new entries, please contact: su.matthew.paul.y3@f.mail.nagoya-u.ac.jp.
